# Challenges in speed and power development across age categories: a comparative study of female and male football players

**DOI:** 10.5114/biolsport.2025.150039

**Published:** 2025-05-14

**Authors:** Irineu Loturco, Pedro E. Alcaraz, Lucas P. Oliveira, Lucas D. Tavares, Bernardo Requena, Tomás T. Freitas, Lucas A. Pereira

**Affiliations:** 1 NAR – Nucleus of High Performance in Sport, São Paulo, Brazil; 2 Department of Human Movement Sciences, Federal University of São Paulo, São Paulo, Brazil; 3 UCAM Research Center for High Performance Sport, UCAM – Universidad Católica de Murcia, Murcia, Spain; 4 Facultad de Deporte, UCAM – Universidad Católica de Murcia, Murcia, Spain; 5 FSI – Football Science Institute; 6 Scientific Department, São Paulo Football Federation, São Paulo, Brazil; 7 SCS – Strength and Conditioning Society

**Keywords:** Athletic performance, Team-sports, Sprint speed, Women athletes, Soccer, Agility

## Abstract

The popularity of women’s football has substantially increased over the last few decades. Despite this growing interest, few studies have compared the evolution of the physical performance of female football players across different age categories, and none have been conducted with players from the same club. Similarly, no studies have compared this progression between female and male players. We compared the evolution of speed-power qualities in female and male players from the same club throughout their specialization process, from the under-20 to the senior category. A total of 101 youth and senior female and male football players performed vertical jump, sprint speed, change-of-direction (COD) speed, and jump-squat power tests in this order, each conducted on separate occasions. Additionally, sprint momentum (SM) and COD-Deficit were also computed. Senior players demonstrated superior countermovement jump performance compared to under-20 players in both groups (ES = 0.86–0.93; P < 0.05). No significant differences were observed in squat jump and jump-squat power between under-20 and senior players (P > 0.05). Similarly, no differences were found in any speed-related tests between under-20 and senior players in either group (P > 0.05). SM and COD-Deficit were similar across age categories for both sexes; however, SM was higher in men (ES ≥ 2.05), while COD-Deficit was lower in female players (ES ≥ 2.08; P < .001). In conclusion, men outperformed women in all physical tests, with smaller differences in speed-related performance (Difference ≤ 14.2%). This gap was even narrower in more complex speed tasks, such as COD drills (Difference ≤ 4.7%), and may be attributed to the significant difference in SM; greater in male players) and its impact on the COD-Deficit.

## INTRODUCTION

The popularity and professionalism of women’s football have substantially increased over the last few decades [1, 2]. In the United Kingdom, for example, a country that has one of the most famous and important men’s football leagues in the world (i.e., the “Premier League”) [3], women’s football is now one of the most popular sports across various team sport disciplines [4]. Indeed, as early as 1995, Joseph Blatter, who was the General Secretary of the International Federation of Association Football (FIFA) at the time, declared: “The future is feminine” [5]. A similar movement has also been noted in other European and American countries (e.g., Norway, Sweden, Brazil, the US, etc.) [6–9], with several studies reporting this rapid expansion. Nevertheless, despite this evident growing trend and interest, women’s football still suffers from a lack of appropriate organization and structure, which certainly impacts the professionalism of the sport and, consequently, of female football players [10]. In this regard, it is not uncommon to find female players who combine their football careers with complementary activities (e.g., studying or other types of professional work) [4, 11].

The same lack of professionalism also affects other areas of women’s football, such as the proper and specific development of sport science and training strategies for this athletic population. A recent scoping review sheds light on this issue by reporting that the most commonly researched topic in women’s football is related to injuries, with the majority of studies involving only elite senior football players [12]. Hence, there is a clear need for more studies describing, for example, the gradual changes and evolution of the physical and technical qualities of female football players during their transition from youth to senior categories – an aspect that has been widely researched in men’s football and other male and female team sports [13–15]. Indeed, previous studies have shown no significant differences in sprint speed and power between younger and senior male football players, although older players may exhibit meaningful decrements in certain speed- and power-related capacities (i.e., acceleration, change-of-direction [COD] speed, and vertical jump ability) as they progress through the age categories [16, 17]. In contrast, in women’s rugby sevens, speed and jump performance demonstrate moderate to large improvements as players advance from junior to elite categories [18]. Furthermore, in rugby sevens, as expected, moderate to large differences (i.e., effect sizes) in 10- and 40-m sprint speed, jump height, and “on-field performance measures” (e.g., top-speed and sprint distance covered during match play) have been observed between male and female players across different playing levels [18]. Taken together, these data could assist coaches in making more informed decisions regarding training practices. As such, a similar study comparing male and female football players by gender and age categories would be highly relevant for practitioners working in women’s football.

Two recent studies compared selected performance measures between youth and senior female football players. In one of these studies, Eustace et al [19] reported that absolute and angle-specific strength measures, assessed via isokinetic dynamometry of the knee flexors and extensors, effectively distinguished between age categories, with peak torque and angle-specific torque being the most useful variables for informing individual training needs, especially in youth players. Doyle et al [20] also examined differences in physical performance among under-17, under-19, and senior National Team Irish female football players and observed no significant changes, either positive or negative, between the age groups in the 10-m, 20-m, and 30-m linear speed measurements. Conversely, Ramos et al [21] reported that senior players are faster over 20 m compared to their younger counterparts from the Brazilian National Teams (i.e., U15, U17, and U20 female football players), while Haugen et al [22] identified a moderate but progressive development in zero to 20-m sprint speed among elite female football players over time, although vertical jumping ability remained stable. Notably, none of these studies specifically focused on youth and senior players from the same club – which is essential to draw more robust conclusions about the hypothetical evolution of physical performance in women’s football – or compared these potential changes with those exhibited by male football players at the same playing levels.

Therefore, the aims of this study were: (1) to test the differences in speed, jump, and power capacities of elite female football players from distinct age categories (i.e., under-20 vs. senior players); (2) to compare the differences and the evolution of these physical qualities between male and female football players, across their prospective development. Due to differences in training background, with male players exposed to higher levels of competitiveness and technical support from an early age [11, 23, 24], we hypothesized that female players would demonstrate greater performance improvements from the under-20 to the senior category.

## MATERIALS AND METHODS

### Subjects

One-hundred and one professional football players participated in this study, categorized as follows: (a) men under-20: n = 32; age: 18.7 ± 1.5 years; height: 1.80 ± 0.08 m; body mass (BM): 76.1 ± 8.3 kg; (b) men senior: n = 18; age: 24.3 ± 4.3 years; height: 1.83 ± 0.08 m; BM: 80.2 ± 7.4 kg; (c) women under-20: n = 27; age: 17.9 ± 4.8 years; height: 1.64 ± 0.07 m; BM: 62.0 ± 8.7 kg; and (d) women senior: n = 24; age: 25.9 ± 5.6 years; height: 1.67 ± 0.06 m; BM: 65.4 ± 8.4 kg. All senior and under-20 players belonged to the same football club, while male and female players were part of different clubs. Both the clubs and categories included in this study competed at the national level. Athletes were assessed at the beginning of their respective preseason periods. The study was approved by the local Ethics Committee, and all subjects provided informed consent before participation.

### Design

This cross-sectional study compared the differences in speed, jump, and power capacities among male and female football players from distinct age categories. Each group followed the same testing protocol, maintaining the same sequence, on separate occasions, according to their training and competition schedules. After a general and specific warm-up including 10 minutes of light-to-moderate running, dynamic stretching, and submaximal attempts at each specific exercise, players performed the following tests: squat jump (SJ), countermovement jump (CMJ), sprinting speed at 5, 10, and 20 m, Zigzag COD test, and peak power in the jump squat exercise (JSPP) using a load equivalent to 60% BM [17]. Sprint momentum (SM) (i.e., the product of sprint speed and BM) and COD Deficit (i.e., the difference in time or speed between a linear sprint and a COD test over an equivalent distance) were also computed [25, 26]. All players were already familiar with the testing procedures due to their professional training routine. The typical weekly training programs of female and male football players during the period of the study are presented in Tables 1 and 2, respectively.

### Procedures

#### Vertical Jump Tests

Vertical jump height was assessed using the SJ and CMJ. In the SJ, players maintained a static position with a ≈90° knee flexion angle for 2 s before jumping, without any preparatory movement. In the CMJ, players executed a downward movement followed by full extension of the lower limbs, with the depth of the countermovement freely determined to avoid changes in jumping coordination [27]. All jumps were performed with hands on the hips. Each player performed five jump attempts, with 15-second intervals between successive trials. The tests were conducted using a contact platform (Elite Jump System; S2 Sports, São Paulo, Brazil), and the best attempt was used for data analysis.

### Sprinting Speed and Momentum

Sprint speed testing was conducted on an indoor running track. Four pairs of photocells (Elite Speed System; S2 Sports, São Paulo, Brazil) were positioned at the starting line and at distances of 5, 10, and 20 m. Players performed two sprints, starting from a standing position 0.5-m behind the first pair of timing gates. Sprint speed was calculated as the distance traveled over a measured time interval. SM (kg · m · s^−1^) was obtained by multiplying the player’s BM by their 20-m linear sprint speed [25]. A 5-min rest interval was allowed between trials, and the fastest trial was considered for analysis.

### Zigzag Change of Direction Test and COD Deficit

The Zigzag COD test consisted of four 5-m sections marked with cones set at 100° angles. Players were required to decelerate and accelerate as quickly as possible around each cone. Two maximal attempts, separated by a 5-min rest, were performed. Starting from a standing position 0.5-m behind the first pair of photocells (Elite Speed, S2 Sports, São Paulo, Brazil), athletes were instructed to complete the test as quickly as possible until crossing the second pair of photocells, placed 20 m from the starting line. The faster of the two trials was used for analysis. The Zigzag COD Deficit was calculated as the difference between the 20-m linear sprint speed and Zigzag COD test speed [25, 26].

### Peak Power in the Jump Squat Exercise

PP was assessed in the JS exercise using a load corresponding to 60% of the athletes’ BM [17]. The test was conducted on a Smith Machine (Hammer Strength Equipment, Rosemont, IL, USA). Players executed three repetitions at maximal intended velocity, with 15-s intervals between successive attempts. To determine PP, a linear velocity transducer (T-Force, Dynamic Measurement System; Ergotech Consulting S.L., Murcia, Spain), sampling at 1000 Hz, was attached to the barbell. The highest PP value recorded was used for analysis. Data were normalized by dividing the absolute PP values by the athletes’ BM (i.e., relative power = W · kg^−1^).

### Statistical Analysis

Data are presented as means ± standard deviation. Data normality was tested using the Shapiro-Wilk test. Comparisons between the four groups of players were performed using a one-way analysis of variance (ANOVA). Tukey’s post-hoc test was used to determine where significant differences occurred. The significance level was set at P < 0.05. Cohen’s *d* [28] effect sizes (ES) were calculated to estimate the magnitude of significant differences and interpreted according to the thresholds proposed by Rhea [21] for highly trained subjects: < 0.25 (trivial), 0.25–0.50 (small), 0.50–1.00 (moderate), and > 1.00 (large).

## RESULTS

All measurements used in this study exhibited high levels of relative and absolute reliability (i.e., ICC > 0.90 and CV < 10%). Table 3 presents the comparisons of vertical jump height between under-20 and senior female and male players. Superior CMJ performance was observed for senior players compared to under-20 players in both male and female groups (P < 0.05). No significant differences were found in SJ and JSPP between under-20 and senior players (P > 0.05). Table 4 displays the comparisons of linear sprint and COD speed performance between under-20 and senior female and male players. No significant differences were detected between under-20 and senior players in either group for any of the speed-related tests (P > 0.05). SM and COD Deficit were similar across age categories for both female and male players; however, SM was significantly greater in men, whereas COD Deficit was significantly lower in female football players in both age categories (P < 0.05).

**TABLE 3:** Comparisons of vertical jump tests and jump-squat peak power (JSPP) between male and female football players within and across different age categories.

		Under-20	Senior	*P-value*	*Effect Size*	*% Dif.*
**SJ (cm)**	**Men**	40.8 ± 4.8	39.3 ± 4.8	0.706	0.24	3.5
	**Women**	30.2 ± 4.3	31.2 ± 3.9	0.871	0.19	3.2
	*P-value*	< .001	< .001			
	*Effect Size*	1.86	1.48			
	*% Dif.*	34.8	26.2			

**CMJ (cm)**	**Men**	43.7 ± 5.0	48.8 ± 4.4	< .001	0.86	11.6
	**Women**	29.7 ± 3.6	34.1 ± 4.1	0.003	0.93	14.7
	*P-value*	< .001	< .001			
	*Effect Size*	2.48	2.80			
	*% Dif.*	47.2	43.2			

**JSPP (W · kg-1)**	**Men**	20.1 ± 2.7	19.7 ± 3.9	0.96	0.11	2.1
	**Women**	17.7 ± 2.8	16.3 ± 2.3	0.313	0.42	7.9
	*P-value*	0.012	0.002			
	*Effect Size*	0.71	0.79			
	*% Dif.*	13.4	20.5			

**Note**: SJ = squat jump; CMJ = countermovement jump; %Dif. = percentage of difference.

**TABLE 4:** Comparisons of linear sprint speed tests, sprint momentum (SM), change-of-direction (COD) speed, and COD Deficit (CODD) between male and female football players within and across different age categories.

		Under-20	Senior	*P-value*	*Effect Size*	*% Dif.*
**Speed 5-m (m · s^-1^)**	**Men**	4.76 ± 0.34	4.73 ± 0.22	0.971	0.10	0.8
	**Women**	4.28 ± 0.24	4.20 ± 0.34	0.769	0.23	1.9
	*P-value*	< .001	< .001							
	*Effect Size*	1.28	1.62							
	*% Dif.*	11.3	12.5							

**Speed 10-m (m · s-1)**	**Men**	5.67 ± 0.26	5.66 ± 0.21	0.999	0.04	0.2
	**Women**	5.06 ± 0.20	4.96 ± 0.26	0.426	0.37	2.0
	*P-value*	< .001	< .001							
	*Effect Size*	2.03	2.53							
	*% Dif.*	12.1	14.2							

**Speed 20-m (m · s-1)**	**Men**	6.70 ± 0.22	6.63 ± 0.23	0.844	0.23	1.0
	**Women**	5.93 ± 0.33	5.85 ± 0.28	0.762	0.19	1.3
	*P-value*	< .001	< .001							
	*Effect Size*	2.38	2.54							
	*% Dif.*	12.9	13.3							

**SM 20-m (kg · m · s-1)**	**Men**	509.4 ± 57.8	531.8 ± 52.8	0.477	0.33	4.4
	**Women**	367.4 ± 54.1	382.5 ± 43.8	0.74	0.24	4.1
	*P-value*	< .001	< .001							
	*Effect Size*	2.05	2.44
	*% Dif.*	38.6	39.1							

**COD Speed (m · s-1)**	**Men**	3.33 ± 0.11	3.34 ± 0.08	0.969	0.11	0.4
	**Women**	3.26 ± 0.13	3.19 ± 0.09	0.15	0.45	2.0
	*P-value*	0.05	< .001							
	*Effect Size*	0.50	1.45							
	*% Dif.*	2.2	4.7							

**CODD (m · s-1)**	**Men**	3.37 ± 0.24	3.29 ± 0.25	0.756	0.27	2.4
	**Women**	2.67 ± 0.32	2.66 ± 0.24	0.999	0.03	0.4
	*P-value*	< .001	< .001							
	*Effect Size*	2.08	2.09							
	*% Dif.*	26.0	23.5							

## DISCUSSION

For the first time, we simultaneously tested and compared the physical performance of female and male football players from the same club across different age categories. Moreover, this is the first study to examine the evolution of power output using the loaded JS, comparing both sexes and age groups. Somewhat surprisingly, and in contrast with our hypothesis, the prospective evolution of speed, jump, and power capacities in elite youth and senior female football players is very similar to the evolution – or even the stagnation – observed in male football players [17, 29, 30]. Therefore, differences in training background do not appear to significantly influence the gradual physical development in women’s football. For both sexes, the increase in training volume and intensity, combined with a more congested competitive schedule, seems to be a key factor affecting chronic training responses [31–33].

As expected, male football players outperformed female players at both playing levels in all speed- and power-related tests [25, 34]. However, certain relative differences in physical performance between sexes warrant further discussion. For example, while the differences in SJ, CMJ, and JSPP were, on average, 31%, 45%, and 17%, respectively, across both age categories, the differences in sprinting speed over distances from 5 to 20 m were all ≤ 13%, with a minimal difference of only 3.5% for COD speed (Tables 3 and 4). To some extent, these results reinforce previous findings suggesting that the potential for development in speed qualities is highly limited, regardless of the sport discipline, age category, training strategy, and sex [17, 29, 35]. In addition, the marginal difference of 3.5% reported for the COD test can be easily justified by previous studies, which indicate that greater SM is associated with higher levels of COD Deficit [36]. As a result, due to their superior sprinting capacity (+ ≈13%) and greater BM (+ ≈22%), male football players also exhibit greater SM (+ ≈40%) (Table 4), thus reducing their ability to effectively utilize their higher speeds for rapid directional changes. Practitioners should take this into account when training football players of both sexes, as heavier and faster players will always display greater SM, which directly and negatively affects their COD performance [25, 36].

Contrary to our hypothesis, the progression and projected development of speed and power qualities in female football players across age categories closely mirrored those observed in male players (Tables 3 and 4) [16, 17]. Despite their limited training background and a lack of proper structure at younger ages, female football players showed no significant differences from their male peers during the transition from the under-20 to the senior category. In men’s football, it is well established that elite players must face and overcome the challenges posed by concurrent training effects throughout their professional careers, which, according to many authors, may compromise the adequate development of certain neuromuscular capabilities [16, 31, 37, 38]. This is because the higher volume and intensity of technical-tactical training, combined with a greater frequency of matches, long journeys, and reduced time for recovery at the senior level, may interfere with the continuous and effective development of physical performance – a factor that becomes even more pronounced toward the end of the season [37, 39]. Although we acknowledge that this factor can also affect female football players, two main reasons justify our initial hypothesis: (1) the limited training background and lack of a professional structure at earlier ages could extend the window for appropriate physical development in female football players, thus facilitating and optimizing their positive responses to training at the senior level, where they typically encounter more structured processes and tournaments [23, 24, 40], and (2) even considering the substantial increase in training and match frequency during the transition from under-20 to the senior level in women’s football (Table 2), the total training volume and number of matches remain lower than those observed in men’s football (Table 1). Nonetheless, these relative increases in training volume and competitiveness experienced by female senior players appear sufficient to mitigate the potential gains in physical performance that could be achieved in a more organized context. Practitioners aiming to enhance the neuromuscular performance of female football players should consider, when feasible, the possibility of “sacrificing” (i.e., reducing) the relative content of football-specific training over a targeted phase [29], thereby prioritizing an increase in the number and frequency of speed and power training sessions during this period. The same procedure is also fundamental for senior male players, who consistently exhibit high stability in their speed performance over the course of their professional careers [16, 17, 29].

**TABLE 1:** Typical weekly training schedule for female under-20 and senior football players.

		Monday	Tuesday	Wednesday	Thursday	Friday
**Under-20**	**Morning**	–	TEC/TAC 60’	–	TEC/TAC 45–60’	TEC/TAC 45–60’

	**Afternoon**	TEC/TAC 45’ OR **RECOVERY SESSION	RT 30’	–	RT 30’	–

**Senior**	**Morning**	–	RT/CT 45’	TEC/TAC 45–60’	RT 45’	TEC/TAC 60’

	**Afternoon**	TEC/TAC 45–60’ OR **RECOVERY SESSION	TEC/TAC 60–70’	–	TEC/TAC 60’	–

**Notes**: *TEC/TAC: technical-tactical training sessions involving specific game actions and small-sided games in different formats; RT: resistance training sessions incorporating traditional and ballistic strength-power exercises with light-to-moderate loads, and plyometric exercises performed under unloaded conditions; CT: core training. **Recovery sessions are routinely conducted after both friendly and official matches.

**TABLE 2:** Typical weekly training schedule for male under-20 and senior football players.

		Monday	Tuesday	Wednesday	Thursday	Friday
**Under-20**	**Morning**	–	TEC/TAC 60–70’	–	TEC/TAC 60–70’	TEC/TAC 60’

	**Afternoon**	RT 30’ + TEC/TAC 45’ OR **RECOVERY SESSION	RT 45’	–	RT 45’	–

**Senior**	**Morning**	–	RT/CT 60’	TEC/TAC 60’	RT 45–60’	TEC/TAC 60–70’

	**Afternoon**	RT 30’ + TEC/TAC 45–60’ OR **RECOVERY SESSION	TEC/TAC 80–90’	–	TEC/TAC 70–80’	–

Notes: *TEC/TAC: technical-tactical training sessions involving specific game actions and small-sided games in different formats; RT: resistance training sessions incorporating traditional and ballistic strength-power exercises with light-to-moderate loads and plyometric exercises performed under unloaded conditions; CT: core training. **Recovery sessions are routinely conducted after both friendly and official matches

In summary, our findings align with previous studies conducted with male and female football players from the same or different clubs, across various age categories, and competing at distinct playing levels. First, the increases in sprint speed over different distances are negligible from the under-20 to the senior category, for both male and female players [16, 17, 20, 29]. Second, the relative differences in speed-related capacities between male and female players (i.e., 3.5% to 13%) are considerably smaller than those observed in power-related capacities (i.e., 17% to 45%). Third, the smallest difference among all physical qualities was recorded in COD speed – an aspect that is clearly understandable when considering the negative impact of SM on the COD Deficit, a phenomenon already detected in other team sports and even in football [25, 36, 41].

This research is limited by its cross-sectional design and the inability to manipulate or adjust training loads and programs during the prospective transition between age categories. Nevertheless, it is worth noting that a series of previous investigations have failed to identify effective strategies for improving the speed performance of elite athletes throughout their specialization process or during specific training phases, regardless of whether they are men or women. Irrespective of their training background, this issue also appears to be a significant barrier for female football players.

## CONCLUSIONS

Men outperformed women in all physical performance tests, with a smaller difference in speed-related performance. This gap is even narrower in more complex speed tasks, such as directional changes, which may be linked to the significant difference in SM (greater in male football players) and its direct influence on COD Deficit [25, 36, 41]. Similarly, except for the CMJ test, male and female players do not exhibit significant differences between age categories in other speed, jump, and power measures (i.e., linear sprints, SJ, and JSPP). This limited progression might be attributed to the effects of concurrent training (i.e., interference effects) [16, 17, 37, 39], commonly associated with the congested training and competition schedules in senior categories. As such, the evolution and prospective development of physical performance – specifically in speed and power abilities – among youth and senior players over the course of their careers remains a considerable challenge for practitioners. This study also provides a reference dataset on speed, jump, and power capacities, offering benchmarks to evaluate the evolution of physical performance in elite female football players across different age categories. Our findings indicate that the prospective evolution of neuromuscular qualities in elite youth and senior female football players closely mirrors that observed in their male counterparts. Therefore, differences in training background do not appear to significantly impact the gradual physical development in women’s football. This insight is especially valuable for practitioners, as it offers guidance on systematically evaluating and monitoring athlete progression, optimizing training programs, and addressing specific related to speed and power development. Further research is warranted to support the continuous evolution of physical performance in both male and female players in elite football.
